# Supplementary material for: Transport in helical Luttinger liquids in the fractional quantum Hall regime
Source: Nat Commun. 2021 Sep 7;12:5312. doi: 10.1038/s41467-021-25631-2 (PMC8423831; doi:10.1038/s41467-021-25631-2)
Supplement: Supplementary file 1 — Supplementary Information [file 41467_2021_25631_MOESM1_ESM.pdf]

Transport in helical Luttinger liquids in the fractional quantum Hall regime

Ying Wang, Vadim Ponomarenko, Kenneth W. West, Kirk Baldwin, Loren N. Pfeiffer, Yuli Lyanda-Geller, Leonid P. Rokhinson

Supplementary Information

CONTENTS

I. Supplementary Figures 2

II. Supplementary Note 1: Devices characterization 3

III. Supplementary Note 2: Transport in the presence of a chiral channel 7

IV. Supplementary Note 3: Modeling of transport through a helical domain wall at $\nu = 2/3$ 9

A. Description of edges of $\nu = 2/3$ state in terms of bosonic fields and quasiparticle bosonic fields 9

B. Description of edges of $\nu = 2/3$ state in terms of separate charge and neutral/spin modes 10

C. The Luttinger liquid action in the presence of both spin-polarized and spin-unpolarized phases. 12

D. Tunneling and charge currents 13

E. Tunneling in the model of zero length hDW 13

F. Ballistic domain wall of finite length with scattering at the ends 16

G. Account for tunneling between the same spin modes in the polarized region 18

H. General case of the domain wall of finite length 19

I. Summary of charge, spin and neutral currents in the absence of spin flip processes 23

J. Effect of spin flip processes 24

References 26

I. SUPPLEMENTARY FIGURES

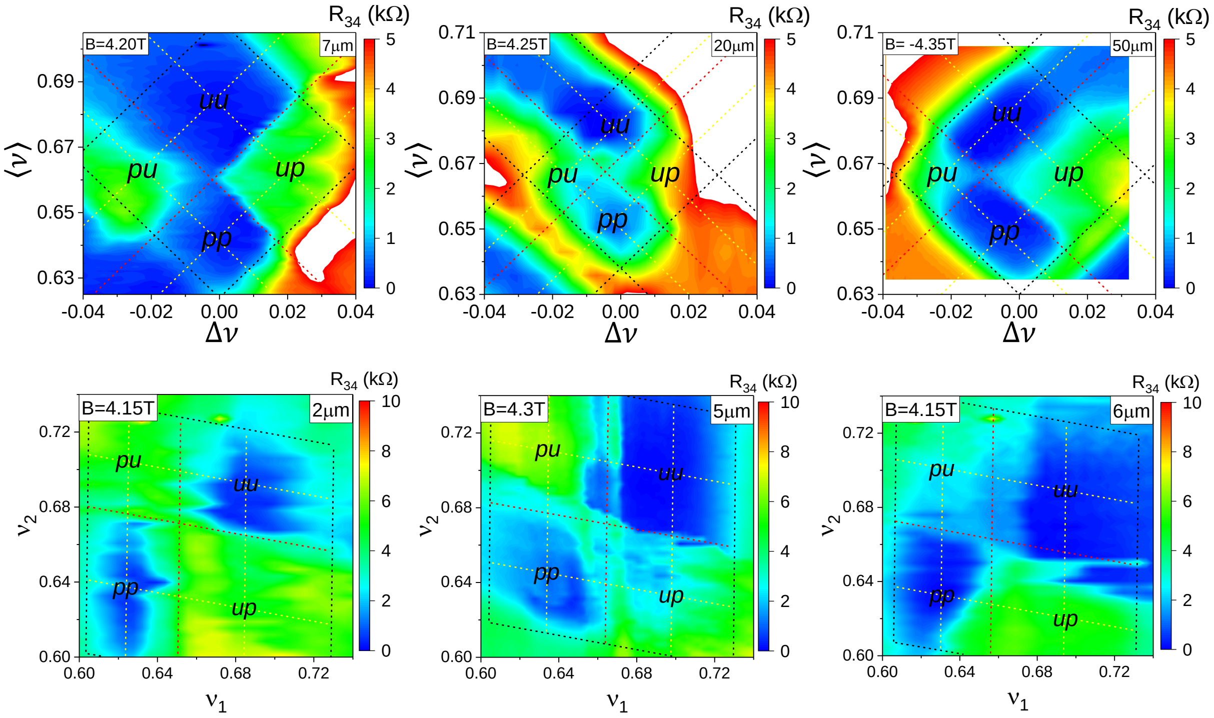

Supplementary Figure 1. **Formation of helical domain walls of different length.** Resistance R_{34} is plotted as a function of $\langle \nu \rangle$ and $\Delta\nu$ for samples with 7 μm, 20 μm and 50 μm gates boundary and as a function of ν_1 and ν_2 for samples with 2 μm, 5 μm and 6 μm gates boundary. Black lines outlines the $\nu = 2/3$ region, red lines mark u-p transitions and yellow lines mark centers of u and p regions.

II. SUPPLEMENTARY NOTE 1: DEVICES CHARACTERIZATION

After cooldown from room temperature to 4 Kelvin devices are illuminated with a red LED at 100 μA for 1 min and left to relax for 12 hours before cooling to the base temperature. This sequence is found to result in the best quality and uniformity of a 2D gas in our experiments, with the sharpest spin transition and widest fractional quantum Hall states. With zero gate voltages densities of 2D gases under gates G1 and G2 are approximately $0.9 \cdot 10^{11}$ cm^{-2}, see Fig. 2. Small differences in the quality of 2D gases under two gates is attributed to different thickness of gate oxides ($50nm$ under G1 and $100nm$ under G2), as well as to a small difference in the thickness of semi-transparent titanium layers which form gate electrodes. Application of negative gate voltage and reduction of the density to $\approx 0.7 \cdot 10^{11}$ cm^{-2} does not degrade quality of 2D gases as shown in Fig. 2b.

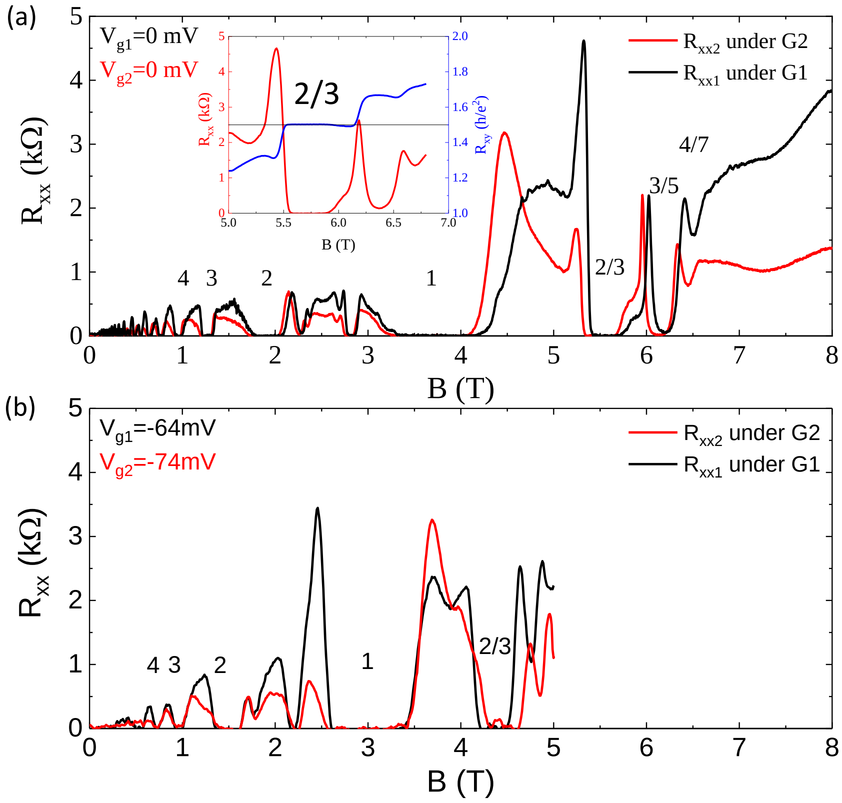

Supplementary Figure 2. **Magnetoresistance and quality of the QHE.** Magnetoresistance R_{xx} of a 2D gas as a function of magnetic field at (a) zero gate voltages and (b) gate voltages where $\nu = 2/3$ is close to the spin transition measured at the base temperature. Inset shows R_{xx1} and R_{xy1} for $V_{g1} = 0$ from the same cooldown but different LED sequence.

Evolution of the 2D gas resistance in the vicinity of $\nu = 2/3$ as a function of magnetic field and gate voltage is shown in Supplementary Fig. 3. Electron density is extracted from the position of $\nu = 3/5$ state (within this field range $\nu = 3/5$ is far from the spin transition). Electron density is a linear function of gate voltages $n(V_g) = n_0 + \beta V_g$, where coefficients $\beta_1 = 4.76 \cdot 10^8 cm^{-2}/mV$ and $\beta_2 = 3.21 \cdot 10^8 cm^{-2}/mV$ are almost the same for different cooldowns while the zero voltage density n_0 varies within 5%.

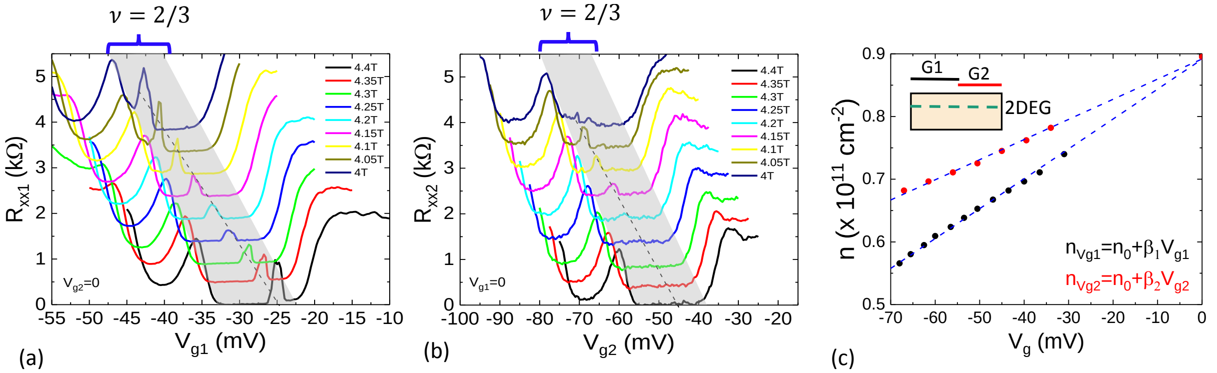

Supplementary Figure 3. **Gate control of the ferromagnetic transition.** Magnetoresistance R_{xx} of a 2D gas under (a) G1 and (b) G2 as a function of gate voltage at different magnetic fields in the vicinity of $\nu = 2/3$. Grey shading outlines the $\nu = 2/3$ state, dashed lines mark the spin transition. (c) Electron density as a function of gate voltage, dashed lines are linear fits. In the insert there is a schematic of the device cross section which shows difference in gate oxide thicknesses.

For the analysis of the domain wall conductance we adjust magnetic field to position spin transitions under both gates to be close to the middle of the $\nu = 2/3$ state, as shown in Supplementary Fig. 4.

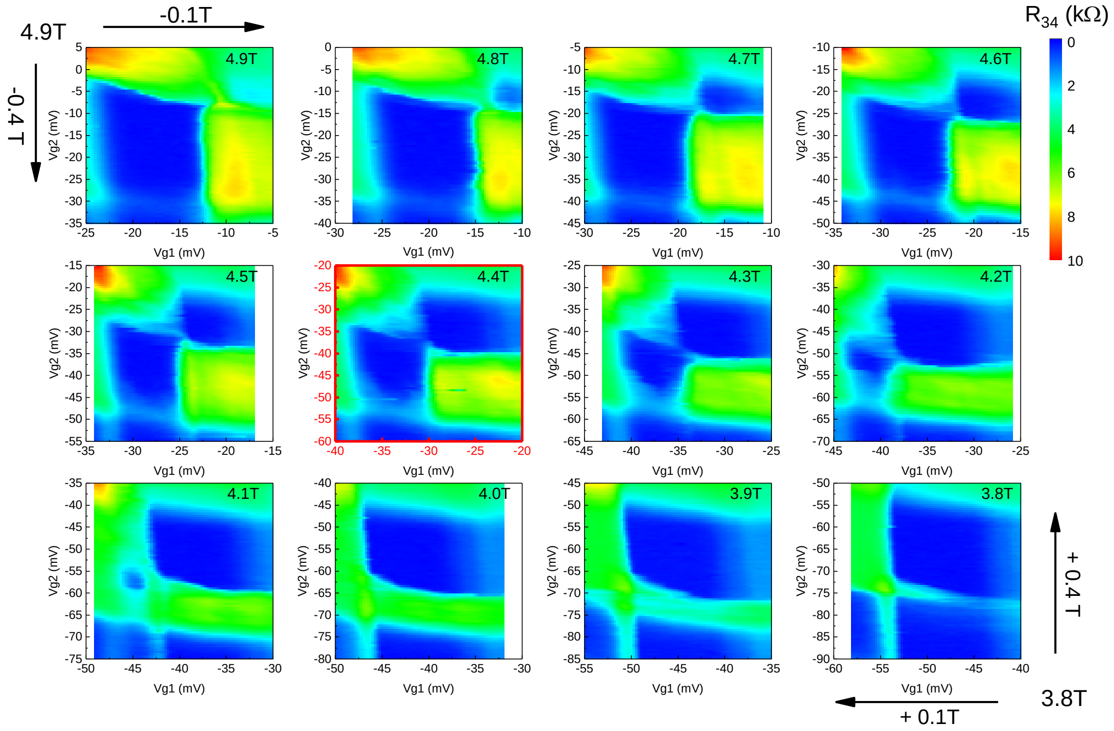

Supplementary Figure 4. **Magnetic field control of the ferromagnetic transition.** Evolution of spin transition boundaries with magnetic filed for a $L = 7$ μm hDW device. Red rectangle marks the field where spin transitions under both gates are centered within the $\nu = 2/3$ state, the optimal field may differ by ± 0.2 T between different cooldowns.

Tuning spin transitions into the middle of the $\nu = 2/3$ state provides wide range of gapped *pp* and *uu* regions, with activation gaps shown in Supplementary Fig. 5. The values of the gaps are extracted from a linear fit to an Arrhenius plot of $R(T)$.

Finally, Supplementary Fig. 6 shows resistance in the vicinity of $\nu = 2/3$ for two opposite field directions. As expected for a helical channel hDW resistance does not depend on the field direction, at least close to the center of the $\nu = 2/3$ state (at the edges of the $\nu = 2/3$ state there is an onset of chiral channels formation, resistance of chiral channels depends strongly on field direction as discussed in more details in the next section).

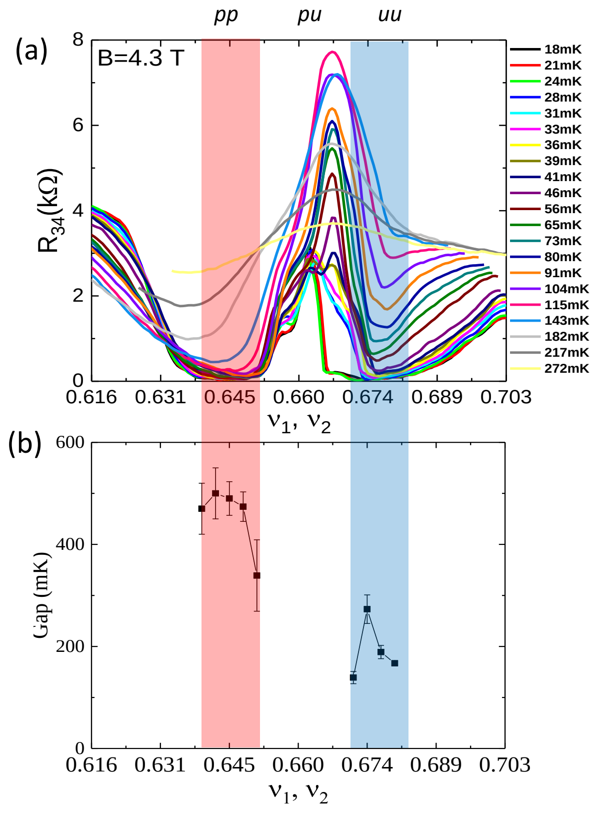

Supplementary Figure 5. **Excitation of excitation gap in the vicinity of the ferromagnetic transition.** (a) Temperature dependence of resistance measured across a $L = 7$ μm hDW for various temperatures. (b) Evolution of an activation gap.

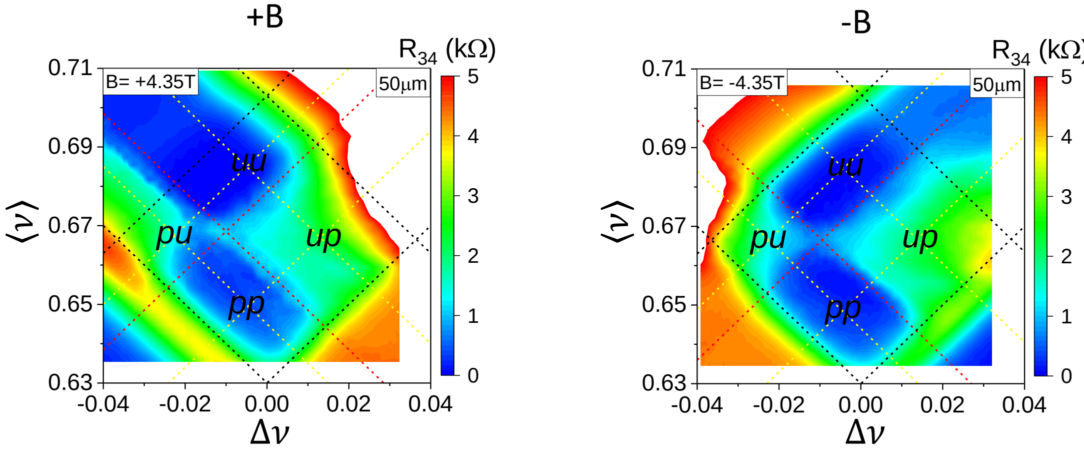

Supplementary Figure 6. **Time inversion symmetry of a hDW.** Stability of hDW resistance R_{34} under magnetic field inversion.

III. SUPPLEMENTARY NOTE 2: TRANSPORT IN THE PRESENCE OF A CHIRAL CHANNEL

In order to contrast helical and chiral channels we perform measurements of R_{34} when filling factors ν_1 and ν_2 under gates $G1$ and $G2$ are different quantum Hall liquids. According to Landauer-Büttiker theory[1,2] for $B > 0$ $R_{34} = (\frac{1}{\nu_1} - \frac{1}{\nu_2}) \cdot R_q$ for $\Delta\nu > 0$ and $R_{34} = 0$ for $\Delta\nu < 0$ (zero and non-zero values will be switched for $B < 0$). In Supplementary Fig. 7abc, resistance R_{34} is plotted over a range of filling factors in IQHE and FQHE regimes. In (d) we plot experimentally measured R_{34} scaled by $(\frac{1}{\nu_1} - \frac{1}{\nu_2})$; the values fall within 1% of the expected values.

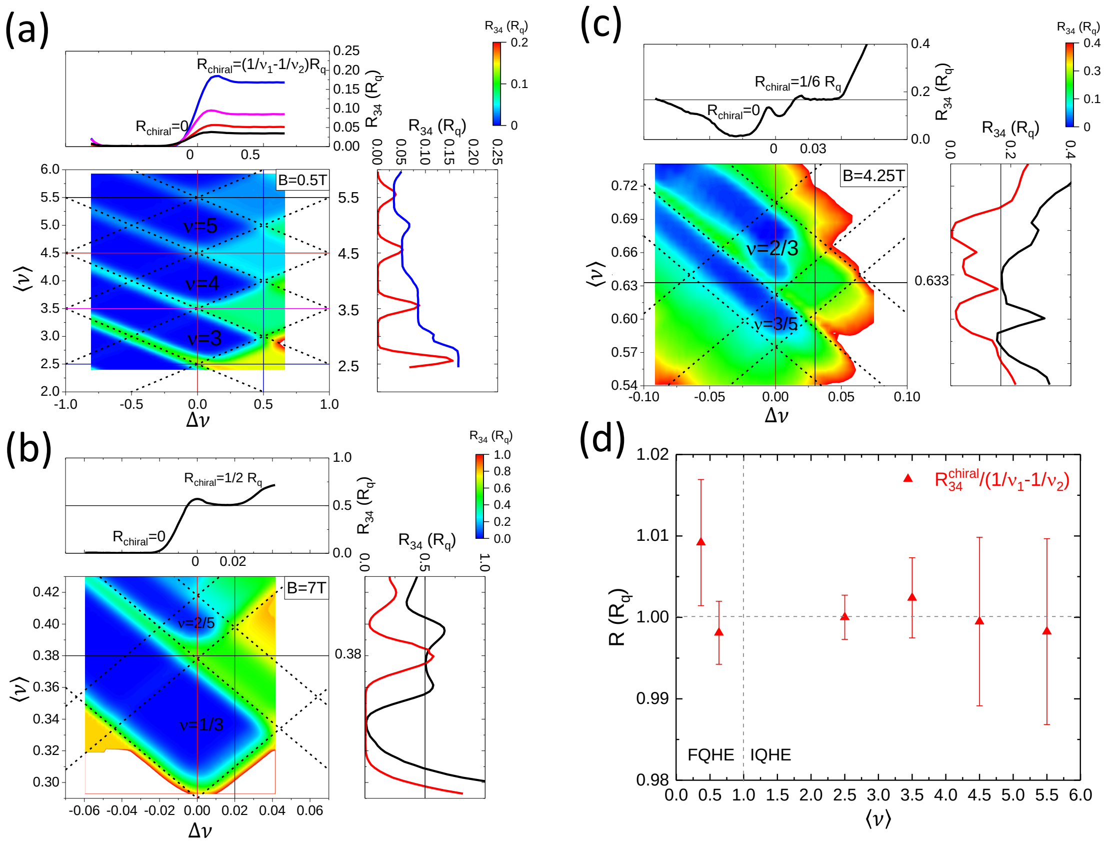

Supplementary Figure 7. **Chiral channels in IQHE and FQHE regimes**. (a-c) Resistance R_{34} is plotted as a function of $\langle\nu\rangle$ and $\Delta\nu$ for the sample with 20 μm gates boundary. The top and right panels in each plot show line cuts indicated in color plots by solid lines. Resistance R_{34} is quantized for $\Delta\nu = +1$ in the IQHE regime and for $\Delta\nu^* = +1$ in the FQHE regime (ν^* is a composite fermion filling factor), $R_{34} = 0$ for $\Delta\nu = -1$ and $\Delta\nu^* = -1$. (d) $R_{34}/(1/\nu_1 - 1/\nu_2)$ for chiral channels is plotted as a function of a filling factor, error bars are standard deviations obtained by averaging R_{34} within $\Delta\nu = \pm 0.003$ around the center of the IQHE or the FQHE state.

IV. SUPPLEMENTARY NOTE 3: MODELING OF TRANSPORT THROUGH A HELICAL DOMAIN WALL AT $\nu = 2/3$

A. Description of edges of $\nu = 2/3$ state in terms of bosonic fields and quasiparticle bosonic fields

Luttinger liquid action for $\nu = 2/3$ edge states in terms of bosonic fields of ***electron operators*** $\Phi_{1,2}$

$$S = -\frac{1}{4\pi}\int dt \int dx \left[\partial_x \Phi \hat{K}^{-1} \partial_t \Phi + \partial_x \Phi \hat{V}^e \partial_x \Phi\right], \tag{1}$$

and the charge density is

$$\rho_c(x) = \frac{1}{2\pi}\left(q\hat{K}^{-1}\partial_x\Phi(x)\right) = \frac{1}{2\pi}\frac{1}{3}\left(\partial_x\Phi_1 + \partial_x\Phi_2\right), \tag{2}$$

where matrix K and vector q are given by

$$\mathbf{K} = \begin{pmatrix} 1 & 2 \\ 2 & 1 \end{pmatrix}, \mathbf{q} = \begin{pmatrix} 1 \\ 1 \end{pmatrix}. \tag{3}$$

For interaction matrix $\hat{V}^e$, we assume that diagonal matrix elements for intra-mode Coulomb interaction is defined by the same matrix element V_1 and off-diagonal matrix elements for inter-mode Coulomb interaction is defined by matrix element V_2,

$$\hat{V}^e = \begin{pmatrix} V_1 & V_2 \\ V_2 & V_1 \end{pmatrix}. \tag{4}$$

As was discussed by Wen[3], equal couplings V_1 of both composite fermion modes are the consequences of the long-range Coulomb interaction.

The commutation relation is

$$[\partial_x\Phi_i(x), \Phi_j(x')] = i2\pi K_{ij}\delta(x - x'). \tag{5}$$

For composite fermion creation operators, we have

$$\Psi_j^\dagger(x) \propto \exp\left(-i\Phi_j(x)\right) \tag{6}$$

and

$$\left[\rho_c(x), \Psi_j^\dagger(x')\right] = \delta(x - x')\Psi_j^\dagger(x'). \tag{7}$$

Luttinger liquid action in terms of ***quasiparticle bosonic fields*** χ , defined by $\Phi = \hat{K}\chi$ is given by

$$S = -\frac{1}{4\pi}\int dt \int dx \left[(\partial_x\chi\hat{K}\partial_t\chi) + (\partial_x\chi\hat{V}^{qp}\partial_x\chi)\right], \tag{8}$$

where

$$\hat{V}^{qp} = \hat{K}\hat{V}^{e}\hat{K}. \tag{9}$$

The two sets of fields are orthogonal:

$$[\partial_x\chi_i(x), \Phi_j(x')] = i2\pi\delta_{ij}\delta(x-x'). \tag{10}$$

The charge mode φ_c, and the neutral mode φ_n are expressed as follows:

$$\varphi_{c,n} = \frac{1}{\sqrt{2}}\left(\chi_1 \pm \chi_2\right), \tag{11}$$

which in vector form reads $(\chi_1, \chi_2)^T = \hat{W}(\varphi_c, \varphi_n)^T$, where the operation T transposes a row vector into a column vector. The transformation matrix $\hat{W}$ is given by

$$\mathbf{W} = \frac{1}{\sqrt{2}}\begin{pmatrix} 1 & 1 \\ 1 & -1 \end{pmatrix}, \tag{12}$$

and the following relations hold

$$\Phi_1 = \chi_1 + 2\chi_2 = \frac{1}{\sqrt{2}}\left(3\varphi_c - \varphi_n\right), \tag{13}$$

$$\Phi_2 = \chi_2 + 2\chi_1 = \frac{1}{\sqrt{2}}\left(3\varphi_c + \varphi_n\right). \tag{14}$$

The neutral mode φ_n can be interpreted as a difference in the occupation of edge modes corresponding to the first and second Λ-levels of composite fermions. In the unpolarized phase it coincides with the spin density and we will use spin index s instead of n.

B. Description of edges of $\nu = 2/3$ state in terms of separate charge and neutral/spin modes

In order to discuss the application of voltage to the domain wall system it is convenient to formulate Luttinger liquid action in terms of separate charge and neutral/spin modes. Using matrix W defined by Supplementary Eq. (12) the transformed matrix K, given by Eq. (3) becomes

$$\mathbf{W^T K W} = \begin{pmatrix} 3 & 0 \\ 0 & -1 \end{pmatrix}. \tag{15}$$

For the transformed matrix $\hat{V}^{qp}$,

$$V_{cn} = W^T \hat{V}^{qp} W, \tag{16}$$

we obtain

$$V_{cn} = \begin{pmatrix} v_c & v_{cn} \\ v_{cn} & v_s \end{pmatrix}, \tag{17}$$

where in terms of couplings V_1 and V_2 in Supplementary Eq.(4), $v_c = 9(V_1+V_2)$, $v_s = (V_1-V_2)$ and off-diagonal terms $v_{cn} = 0$ as a result of our choice of equal diagonal couplings V_1 of both modes. This allows separation of charge and neutral modes in polarized phase and charge and spin modes for non-polarized phase. As was discussed by Wen[3], in the absence of the mechanism of electron scattering on impurities, this separation in both phases originate from equal couplings V_1 of both composite fermion modes Supplementary Eq. (4) are the consequences of the long-range Coulomb interaction.

In order to distinguish Luttinger liquid modes in p and u phases, we characterize all modes, including bosonic electron modes, quasiparticle modes, and separated charge and neutral (spin) modes by indices p and u. The transformed action for the p phase is

$$S = \frac{1}{4\pi} \int dt \int dx \left[-3\partial_x \varphi_{pc} (\partial_t + v_c \partial_x) \varphi_{pc} + \partial_x \varphi_{pn} (\partial_t - v_n \partial_x) \varphi_{pn} \right]. \tag{18}$$

This action coincides with the one expressed in terms of the charge and neutral fields (Supplementary Eqs. (7)-(8)) in the seminal paper by Kane, Fisher and Polchinsky[4], in the case if no electron scattering that leads to the composite fermion tunneling between the different modes and no coupling between modes takes place. The Luttinger liquid action in u phase is similar and is presented in the main text, Eq. (1), with spin modes entering instead of neural modes. In both phases, charge and neutral, or charge and spin modes separate.

The commutation relations for separated charge and neutral modes in the p phase are given by

$$[\partial_x \varphi_{pc}(x), \varphi_{pc}(x')] = i\frac{2\pi}{3}\delta(x - x'), \tag{19}$$

$$[\partial_x \varphi_{pn}(x), \varphi_{pn}(x')] = -i2\pi\delta(x - x'). \tag{20}$$

In the u phase, the commutation relatons for separated charge and spin modes are given by these equations with substitution $pc \to uc$, $pn \to us$. To acquire non-zero average charge

density and current, density of the charge mode φ_{pc} is shifted, $\varphi_{pc} \to \varphi_{pc}(x,t) + \bar{\varphi}_{pc}$. A non-zero average appears due to a charge current injection,

$$\bar{\varphi}_{pc} = \frac{e\sqrt{2}}{3\hbar}\left(\frac{x}{v_c} - t\right)V, \tag{21}$$

where V is the applied voltage. Then the average current carried by the edge is

$$\bar{j} = -\frac{e}{\sqrt{2}\pi}\partial_t\varphi_{pc} = \frac{e^2}{2\pi\hbar}\frac{2}{3}V. \tag{22}$$

The shift of the charge mode density is described by an addition to the Luttinger liquid action

$$\Delta S = \frac{eV}{4\pi\hbar v_c}\int dt \int dx\sqrt{2}\left(\partial_t + v_c\partial_x\right)\varphi_{pc}, \tag{23}$$

so that $S(\varphi_{pc}) + \Delta S(\varphi_{pc}) = S(\varphi_{pc} - \bar{\varphi}_{pc})$. The case of injection from the unpolarized phase into polarized phase, when φ_{uc} is shifted due to applied voltage instead of φ_{pc}, is described by Supplementary Eqs. (21-23) with the change $pc \to uc$.

C. The Luttinger liquid action in the presence of both spin-polarized and spin-unpolarized phases.

The Luttinger liquid action for the edge states at $\nu = 2/3$ consisting of the two phases, polarized p and unpolarized u, is given by

$$S = -\tfrac{1}{4\pi}\int dt \int dx \left[(\partial_x\Phi_u, \partial_x\Phi_p)\hat{\mathcal{K}}^{-1}\begin{pmatrix}\partial_t\Phi_u\\ \partial_t\Phi_p\end{pmatrix} + (\partial_x\Phi_u, \partial_x\Phi_p)\hat{\mathcal{V}}\begin{pmatrix}\partial_x\Phi_u\\ \partial_x\Phi_p\end{pmatrix}\right], \tag{24}$$

where 4x4 matrices

$$\hat{\mathcal{K}} = \begin{pmatrix}-\hat{K} & 0\\ 0 & \hat{K}\end{pmatrix}, \tag{25}$$

with matrix $\hat{K}$ defined by Supplementary Eq. (3) and

$$\hat{\mathcal{V}} = \begin{pmatrix}\hat{V}^e & 0\\ 0 & \hat{V}^e\end{pmatrix}, \tag{26}$$

with matrix $\hat{V}^e$ is defined by Supplementary Eq. (4). For convenience, in order to keep the form of relations for the current as described above for both p and u phases, we reverse the sign of the quasiparticle field $\chi_u \to -\chi_u$, so that $\Phi_u = K\chi_u$.

D. Tunneling and charge currents

The point contact (junction) $x = 0$ electron tunneling between polarized and unpolarized phases is described by the tunnel Hamiltonian

$$\begin{aligned} \mathcal{H}_T &= -\tilde{t}\cos\left(\Phi_{p1}(0,t) - \Phi_{u1}(0,t)\right) \\ &= -\tilde{t}\cos\tfrac{1}{\sqrt{2}}(3\varphi_{pc}(0,t) - \varphi_{pn}(0,t) - 3\varphi_{uc}(0,t) + \varphi_{us}(0,t)), \end{aligned} \tag{27}$$

where the neutral mode in the unpolarized phase is a spin mode describing the difference in spin density between modes.

The tunneling charge current is given by a shift in φ_{pc} due to the applied voltage V described by Supplementary Eqs. (21, 23):

$$j_T = -\partial_t \hat{Q}_P(t) = i\left[\int dx \rho_{pc}, \mathcal{H}_T\right] = -\tilde{t}\sin\left(\Phi_{p1}(0,t) - \Phi_{u1}(0,t)\right), \tag{28}$$

where $\rho_{pc} = \frac{1}{\sqrt{2}\pi}\partial_x \varphi_{pc}$ is the charge density.

E. Tunneling in the model of zero length hDW

In the strong coupling limit the tunneling current can be found by imposing boundary conditions. A zero-length hDW is the limit $x_1 = x_2 = 0$ of the model of the hDW shown in Fig. 3a of the main text. In order to formulate the boundary conditions, we use quasiparticle modes described by Supplementary Eqs. (13, 14) for polarized and unpolarized liquid. Imposing $\mathcal{H}_T = -\tilde{t}\cos\left(\Phi_{p1}(0,t) - \Phi_{u1}(0,t)\right)$ at $\tilde{t} \to \infty$ as a boundary condition that leads to a jump in the tunneling mode and a continuity in the orthogonal, non-tunneling mode, we obtain:

$$\Phi_{p1}(-0) - \Phi_{u1}(+0) = -(\Phi_{p1}(+0) - \Phi_{p1}(-0)), \tag{29}$$

$$\Phi_{p1}(-0) + \Phi_{u1}(+0) = \Phi_{p1}(+0) + \Phi_{u1}(-0). \tag{30}$$

Using the expressions for quasiparticle modes, Supplementary Eq. (13), we have

$$3\varphi^{\rightarrow}_{pc}(-0) - \varphi^{\leftarrow}_{pn}(+0) = 3\varphi^{\leftarrow}_{uc}(-0) - \varphi^{\rightarrow}_{us}(+0), \tag{31}$$

$$3\varphi^{\leftarrow}_{uc}(+0) - \varphi^{\rightarrow}_{us}(-0) = 3\varphi^{\rightarrow}_{pc}(+0) - \varphi^{\leftarrow}_{pn}(-0). \tag{32}$$

We obtain two more equations defining boundary conditions by imposing them on the two modes orthogonal to the modes described by Supplementary Eq. (13). For these modes

we have

$$\chi_{u2}(x) = \tfrac{1}{\sqrt{2}}(\varphi_{uc}(x) - \varphi_{us}(x)), \tag{33}$$

$$\chi_{p2}(x) = \tfrac{1}{\sqrt{2}}(\varphi_{pc}(x) - \varphi_{pn}(x)), \tag{34}$$

and the boundary conditions for these modes are

$$\varphi_{uc}^{\leftarrow}(-0) - \varphi_{us}^{\rightarrow}(-0)) = \varphi_{uc}^{\leftarrow}(+0) - \varphi_{us}^{\rightarrow}(+0)), \tag{35}$$

$$\varphi_{pc}^{\rightarrow}(-0) - \varphi_{pn}^{\leftarrow}(-0)) = \varphi_{pc}^{\rightarrow}(+0) - \varphi_{pn}^{\leftarrow}(+0)). \tag{36}$$

Using explicit expressions for the quasiparticle modes, we obtain that Supplementary Eqs.(30,36) result in the following four equations defining the outgoing fields via the incoming fields:

$$\begin{aligned}
4\varphi_{pc}^{\rightarrow}(+0) &= 3\phi_{uc}^{\leftarrow}(+0) - \varphi_{us}^{\rightarrow}(-0) + \varphi_{pc}^{\rightarrow}(-0) + \varphi_{pn}^{\leftarrow}(+0), \\
4\varphi_{pn}^{\leftarrow}(-0) &= 3\varphi_{pc}^{\rightarrow}(-0) + 3\varphi_{pn}^{\leftarrow}(+0) - 3\varphi_{uc}^{\leftarrow}(+0) + \varphi_{us}^{\rightarrow}(-0), \\
4\varphi_{uc}^{\leftarrow}(-0) &= 3\varphi_{pc}^{\rightarrow}(-0) - \varphi_{pn}^{\leftarrow}(+0) + \varphi_{uc}^{\leftarrow}(+0) + \varphi_{us}^{\rightarrow}(-0), \\
4\varphi_{us}^{\rightarrow}(+0) &= 3\varphi_{uc}^{\leftarrow}(+0) + 3\varphi_{us}^{\rightarrow}(-0) - 3\varphi_{pc}^{\rightarrow}(-0) + \varphi_{pn}^{\leftarrow}(+0).
\end{aligned} \tag{37}$$

The current injected into the polarized phase due to the applied voltage V shifts only the incoming field

$$\varphi_{pc}^{\rightarrow}(-0) \to \varphi_{pc}^{\rightarrow}(-0) - \frac{\sqrt{2}}{3} eVt/\hbar. \tag{38}$$

Using Supplementary Eq.(37), we obtain that this change leads to the following changes in outgoing fields:

$$\begin{aligned}
\varphi_{pc}^{\rightarrow}(+0) &\to \varphi_{pc}^{\rightarrow}(+0) - \tfrac{1}{4}\tfrac{\sqrt{2}}{3} eVt/\hbar, \\
\varphi_{pn}^{\leftarrow}(-0) &\to \varphi_{pn}^{\leftarrow}(-0) - \tfrac{3}{4}\tfrac{\sqrt{2}}{3} eVt/\hbar, \\
\varphi_{uc}^{\leftarrow}(-0) &\to \varphi_{uc}^{\leftarrow}(-0) - \tfrac{3}{4}\tfrac{\sqrt{2}}{3} eVt/\hbar, \\
\varphi_{us}^{\rightarrow}(+0) &\to \varphi_{us}^{\rightarrow}(+0) + \tfrac{3}{4}\tfrac{\sqrt{2}}{3} eVt/\hbar.
\end{aligned} \tag{39}$$

Describing the currents, we will use indices p and u for the currents on the polarized and unpolarized side, correspondingly. Upper indices *in*, *out* correspond to the incoming and outgoing currents, lower indices c, n and s correspond to charge, neutral and spin currents. On the polarized side, charge and spin currents coincide; on the unpolarized side, neutral and spin currents coinside The average incoming or outgoing current due to quasiparticle

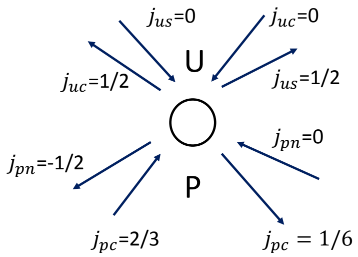

Supplementary Figure 8. **Incoming and outgoing currents in a single point contact.** Current values are in units of $\sigma_0 V$.

modes $\varphi_{\alpha\beta}$, where index α takes values $p.u$ and index β runs over c, n, s is given by the equation

$$j^{in,out}_{\varphi_{\alpha\beta}} = -\frac{e}{2\pi} q a_{\alpha\beta} \sqrt{2} \partial_t \varphi^{in,out}_{\alpha\beta}, \tag{40}$$

where for our choice of signs in action Supplementary Eq. (24) charges $q_p = 1 = -q_u$, q_p is the quasiparticle charge in the polarized phase and q_u is the quasiparticle charge in the unpolarized phase, and coefficients $a_{pc} = a_{uc} = 1$, $a_{pn} = a_{us} = -1$ reflect counting direction of currents in the direction of chirality of modes rather than from the left to the right. We will assume first that the externally induced incoming charge current comes from the contact with potential V to the polarized liquid, and the current flows into the grounded ($V = 0$) contact to the unpolarized liquid. Then the incomig current due to Supplementary Eq. (38) is

$$j^{in}_{pc} = \sigma_0 \frac{2}{3} V \tag{41}$$

and the outgoing currents are given by

$$j^{out}_{pc} = \sigma_0 \tfrac{1}{6} V \tag{42}$$

$$j^{out}_{pn} = -\sigma_0 \tfrac{1}{2} V \tag{43}$$

$$j^{out}_{uc} = +\sigma_0 \tfrac{1}{2} V \tag{44}$$

$$j^{out}_{us} = \sigma_0 \tfrac{1}{2} V, \tag{45}$$

where $\sigma_0 = e^2/h$ is the conductance quantum.

Using the voltage-induced shift of the incoming and outgoing bosonic fields, φ_{pc}, φ_{ps}, φ_{uc} and φ_{us}, given by Supplementary Eqs (38), (39), we can analyze the shift of quasiparticle edge state fields $\chi_{p(1,2)} = \frac{1}{\sqrt{2}}(\varphi_{pc} \pm \varphi_{pn})$ and $\chi_{u(1,2)} = \frac{1}{\sqrt{2}}(\varphi_{uc} \pm \varphi_{us})$, that describes the

distribution of current over the two quasiparticle modes. The calculation shows that the resulting currents associated with these modes are given by

$$j_{p1} = \sigma_0 V \left[\tfrac{7}{12}\theta(-x) + \tfrac{1}{12}\theta(x)\right] \tag{46}$$

$$j_{p2} = \sigma_0 \tfrac{1}{12} V \tag{47}$$

$$j_{u1} = \sigma_0 V \left[-\tfrac{1}{4}\theta(-x) + \tfrac{1}{4}\theta(x)\right] \tag{48}$$

$$j_{u2} = \sigma_0 \tfrac{1}{4} V, \tag{49}$$

where θ-function $\theta(x) = 1$ at $x > 0$ and $\theta(x) = 0$ at $x < 0$ is used to describe incoming and outgoing modes in a single equation. We observe that it follows from these equations that tunneling occurs only between χ_{p1} and χ_{u1} edges, and tunneling charge current is given by

$$j_T = \sigma_0 V(\frac{2}{3} - \frac{1}{6}) = \frac{1}{2}\sigma_0 V. \tag{50}$$

In contrast, states χ_{p2} and χ_{u2} flow, correspondingly, in the polarized and unpolarized region. Modes χ_{p1} and χ_{u1} include modes with the same spin up, while modes χ_{p2} and χ_{u2} carry the opposite spin. This is a consequence of our tunnel Hamiltonian allowing only transmission of like spins. Generalization of the model permitting tunneling with a spin flip, e.g,. induced by interaction with nuclear spins, makes possible some tunneling processes between χ_{p2} and χ_{u2} modes. We will consider modification of the current flow in the presence of spin flips in the subsection J below.

F. Ballistic domain wall of finite length with scattering at the ends

We now analyse a system of two point scatterers at points x_1 and x_2 separated by a ballistic domain wall of a finite length $L = x_2 - x_1$. In the experimental setting, these scatterers are the tri-junctions between edge modes in the p phase, the u phase and modes in the domain wall.

Each of the scatterers is described by Supplementary Eq. (37). To calculate the transmission through two junctions, it is convenient to present the connection Supplementary Eq. (37) between 4-vector of incoming modes $\varphi^{in} = (\varphi^{\rightarrow}_{pc}(x-0), \varphi^{\rightarrow}_{us}(x-0), \varphi^{\leftarrow}_{uc}(x+0), \varphi^{\leftarrow}_{pn}(x+0))^T$ and 4-vector of outgoing modes $\varphi^{out} = (\varphi^{\rightarrow}_{pc}(x+0), \varphi^{\rightarrow}_{us}(x+0), \varphi^{\leftarrow}_{uc}(x-0), \varphi^{\leftarrow}_{pn}(x-0))^T$ in a matrix form:

$$\varphi^{out} = \mathcal{P}\varphi^{in}, \tag{51}$$

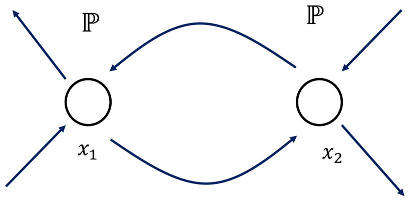

Supplementary Figure 9. **Incoming and outgoing modes in the case of two junctions.** The two junctions are positioned at coordinates x_1 and x_2, describing the total transfer matrix T. Each junction is described by the propagation matrix $\mathcal{P}$ given by the Supplementary Eq. (52) for the zero-length hDW.

where 4x4 matrix $\mathcal{P}$ is given by

$$\mathcal{P} = \begin{pmatrix} P_{++} & P_{+-} \\ P_{-+} & P_{--} \end{pmatrix}, \tag{52}$$

and 2x2 matrices P_{ij},$i, j = \pm$ are defined by

$$P_{++} = P_{--} = \frac{1}{4} \begin{pmatrix} 1 & -1 \\ -3 & 3 \end{pmatrix}, \tag{53}$$

$$P_{+-} = P_{-+} = \frac{1}{4} \begin{pmatrix} 3 & 1 \\ 3 & 1 \end{pmatrix}. \tag{54}$$

The matrix P_{++} describes propagation of chiral modes through a single zero-length scatterer, and the matrix P_{+-} describes the reflection of chiral modes. The total 4x4 transfer matrix $\mathcal{T}$ connects incoming and outgoing modes of two scatterers, via

$$\begin{pmatrix} \Delta_V \varphi^{\rightarrow}_{pc}(x_2+0) \\ \Delta_V \varphi^{\rightarrow}_{us}(x_2+0) \\ \Delta_V \varphi^{\leftarrow}_{uc}(x_1-0) \\ \Delta_V \varphi^{\leftarrow}_{pn}(x_1-0) \end{pmatrix} = \mathcal{T} \begin{pmatrix} \Delta_V \varphi^{\rightarrow}_{pc}(x_1-0) \\ \Delta_V \varphi^{\rightarrow}_{us}(x_1-0) \\ \Delta_V \varphi^{\leftarrow}_{uc}(x_2+0) \\ \Delta_V \varphi^{\leftarrow}_{pn}(x_2+0) \end{pmatrix}, \tag{55}$$

where x_1 and x_2 are positions of the scatterers and Δ_V denotes a voltage-dependent shift of the corresponding field.

Matrix $\mathcal{T}$ is defined by propagation of modes through the scatterers, and potentially by multiple reflections of modes between them. However, as a result of identity

$$P_{++}P_{+-} = 0, \tag{56}$$

the total transfer matrix for the two-junction system is defined only by a single act of reflection, $T_{-+} = P_{-+}$ or by a single propagation through both scatterers, $T_{++} = P_{++}P_{++} = P_{++}$, while any contribution from processes involving subsequent propagations and reflections from scatterers vanishes due to disentanglement property of chiral channels described by Eq. (56).

G. Account for tunneling between the same spin modes in the polarized region

We now consider whether tunneling between the modes with the same spin in the polarized region between two zero length scatterers will change transmission through the domain wall. The boundary condition introduced by the tunneling Hamiltonian corresponding to this process

$$\mathcal{H}_T = -\tilde{t}_p \cos\left(\Phi_{p1}(x) - \Phi_{p2}(x)\right) = -\tilde{t}_p \cos\frac{1}{\sqrt{2}}\varphi_{pn}(x), \tag{57}$$

where $x_1 < x < x_2$, in the strong coupling limit $\tilde{t}_p \to \infty$ is

$$\varphi_{pn}(x-0) = -\varphi_{pn}(x+0). \tag{58}$$

Taking into account this process amounts to changing one of the $\mathcal{P}$ matrices in the model with two scatterers

$$\mathcal{P}_1 = \mathcal{P}\begin{pmatrix} 1 & 0 & 0 & 0 \\ 0 & 1 & 0 & 0 \\ 0 & 0 & 1 & 0 \\ 0 & 0 & 0 & -1 \end{pmatrix}. \tag{59}$$

We then observe that $P_{1++} = P_{++}$, $P_{1-+} = P_{-+}$, as described by Supplementary Eqs. (53,54) and

$$P_{1--} = \frac{1}{4}\begin{pmatrix} 1 & 1 \\ -3 & -3 \end{pmatrix}, \tag{60}$$

$$P_{1+-} = \frac{1}{4}\begin{pmatrix} 3 & -1 \\ 3 & -1 \end{pmatrix}. \tag{61}$$

Therefore, disentangling relations

$$P_{++}P_{1+-} = 0 \tag{62}$$

$$P_{--}P_{1-+} = 0 \tag{63}$$

$$P_{-+}P_{1++} = 0 \tag{64}$$

$$P_{+-}P_{1--} = 0 \tag{65}$$

hold, and no contributions from processes with consequent propagation and reflection occur in the total transfer matrix in the presence of $P1 \to P2$ scattering. The total transfer matrix $\mathcal{T}_c$ for this case is also defined by

$$T_{c++} = P_{++} \tag{66}$$

$$T_{c+-} = P_{+-} \tag{67}$$

$$T_{c-+} = P_{-+} \tag{68}$$

$$T_{c--} = P_{--} \tag{69}$$

It means that $\mathcal{T}_c = \mathcal{P}$ despite scattering in the same spin channel in the polarized region. That leads us to the conclusion that localization and backscattering by the domain wall that results in length-dependent resistance requires spin flips or inelastic processes. We also note that in the Φ_{1p}, Φ_{1u}, χ_{2p}, χ_{2u} representation, only χ_2 modes of opposite spin propagate along the domain wall, while Φ modes having the same spin pass along the edges.This directly correlates with the $\mathcal{T}_c$ matrix properties discussed in the present section.

H. General case of the domain wall of finite length

Here we demonstrate that the coincidence of the currents flowing outside the domain wall in the cases of single-junction, two junctions, as well as two junctions with scatterers in between follows from the imposed strong coupling boundary conditions in a general case of the domain wall of finite length. At the same time, inside the domain wall, the chiral evolution of modes is controlled by the average voltage shifts at their corresponding boundaries.

In the representation of modes Φ_{u1}, χ_{u2}, Φ_{p1}, χ_{p2}, the kinetic matrix $\mathcal{K}_x$ has the form

$$\hat{\mathcal{K}}_x = \begin{pmatrix} -\hat{K}_x & 0 \\ 0 & \hat{K}_x \end{pmatrix}, \tag{70}$$

where

$$\hat{K}_x = \begin{pmatrix} 1 & 0 \\ 0 & -3 \end{pmatrix}. \tag{71}$$

We write the tunneling Hamiltonian as

$$\mathcal{H}_T = -U_T \int_0^L \cos\left(\Phi_{1p}(x) - \Phi_{1u}(x)\right) dx. \tag{72}$$

Here the tunneling constant U_T has dimensions of energy/length, in contrast to tunneling constant $\tilde{t}$ describing point contact/junction tunneling introduced above that has dimensions of energy. At $U_T \to \infty$, the Φ_1 fields become fixed, and there remains the free chiral evolution of the quasiparticle fields χ_{u2}, χ_{p2} described by the action for u and p modes

$$S_u^{DW} = -\frac{3}{4\pi} \int dt \int_0^L dx \partial_x \chi_{u2} \left(\partial_t + v_{DW} \partial_x\right) \chi_{u2}(x,t) \tag{73}$$

$$S_p^{DW} = -\frac{3}{4\pi} \int dt \int_0^L dx \partial_x \chi_{p2} \left(-\partial_t + v_{DW} \partial_x\right) \chi_{p2}(x,t) \tag{74}$$

with the charges $q_{u2}^x = 1 = -q_{p2}^x$ and relations

$$\chi_{u2}(0) = \frac{1}{\sqrt{2}} \left(\varphi_{uc}(0) - \varphi_{us}(0)\right) \tag{75}$$

$$\chi_{p2}(0) = \frac{1}{\sqrt{2}} \left(\varphi_{pc}(0) - \varphi_{pn}(0)\right) \tag{76}$$

$$\chi_{u2}(L) = \frac{1}{\sqrt{2}} \left(\varphi_{uc}(L) - \varphi_{us}(L)\right) \tag{77}$$

$$\chi_{p2}(L) = \frac{1}{\sqrt{2}} \left(\varphi_{pc}(L) - \varphi_{pn}(L)\right) \tag{78}$$

$$\Phi_{p1}(0) = \Phi_{u1}(0) \tag{79}$$

$$\Phi_{p2}(L) = \Phi_{u2}(L). \tag{80}$$

These equations, as in the above sections, reflect the fact that the neutral mode in the unpolarized region coincides with the spin current mode. In the polarized region, spin and charge currents coincide. Boundary conditions for the voltage-dependent average shifts of the modes describe the continuity of the polarized and unpolarized components of the free chiral mode χ_2

$$\Delta_v \bar{\varphi}_{uc}^{\leftarrow}(0) - \Delta_v \bar{\varphi}_{us}^{\rightarrow}(0) = \Delta_v \bar{\varphi}_{uc}^{\leftarrow}(L) - \Delta_v \bar{\varphi}_{us}^{\rightarrow}(L) \tag{81}$$

$$\Delta_v \bar{\varphi}_{pc}^{\rightarrow}(0) - \Delta_v \bar{\varphi}_{pn}^{\leftarrow}(0) = \Delta_v \bar{\varphi}_{pc}^{\rightarrow}(L) - \Delta_v \bar{\varphi}_{pn}^{\leftarrow}(L), \tag{82}$$

and boundary conditions due to tunneling of like spins given by Supplementary Eq. (72) in the strong coupling limit

$$3\Delta_v \bar{\varphi}_{pc}^{\rightarrow}(0) - \Delta_v \bar{\varphi}_{pn}^{\leftarrow}(0) = 3\Delta_v \bar{\varphi}_{uc}^{\leftarrow}(0) - \Delta_v \bar{\varphi}_{us}^{\rightarrow}(0) \tag{83}$$

$$3\Delta_v \bar{\varphi}_{uc}^{\leftarrow}(L) - \Delta_v \bar{\varphi}_{us}^{\rightarrow}(L) = 3\Delta_v \bar{\varphi}_{pc}^{\rightarrow}(L) - \Delta_v \bar{\varphi}_{pn}^{\leftarrow}(L) \tag{84}$$

The first two boundary conditions Supplementary Eqs. (81,82) match the single junction (point contact) conditions Supplementary Eqs. (35,36) resulting from the continuity of χ_{u2},

χ_{p2} across $x = 0$:

$$\Delta_v \bar{\chi}_{u2}(-0) = \Delta_v \bar{\chi}_{u2}(+0) \tag{85}$$

$$\Delta_v \bar{\chi}_{p2}(-0) = \Delta_v \bar{\chi}_{p2}(+0) \tag{86}$$

and coincide with those at $+0 \to L$.

The two other point contact (junction) boundary conditions with $+0$ changed into L read:

$$3\Delta_v \bar{\varphi}^{\rightarrow}_{pc}(0) - \Delta_v \bar{\varphi}^{\leftarrow}_{pn}(L) = 3\Delta_v \bar{\varphi}^{\leftarrow}_{uc}(0) - \Delta_v \bar{\varphi}^{\rightarrow}_{us}(L) \tag{87}$$

$$3\Delta_v \bar{\varphi}^{\leftarrow}_{uc}(L) - \Delta_v \bar{\varphi}^{\rightarrow}_{us}(0) = 3\Delta_v \bar{\varphi}^{\rightarrow}_{pc}(L) - \Delta_v \bar{\varphi}^{\leftarrow}_{pn}(0). \tag{88}$$

The difference of expressions given by Supplementary Eq. (83) and Supplementary Eq. (87) is equal to the difference of expressions given by Supplementary Eq. (84) and Supplementary Eq. (88)

$$\Delta_v \bar{\varphi}^{\leftarrow}_{pn}(L) - \Delta_v \bar{\varphi}^{\leftarrow}_{pn}(0) = \Delta_v \bar{\varphi}^{\rightarrow}_{us}(L) - \Delta_v \bar{\varphi}^{\rightarrow}_{us}(0). \tag{89}$$

This is an identity due to conservation of the spin current in unpolarized phase and the neutral current in the polarized phase. Therefore, the relation between the averages $\Delta\bar{\varphi}^{in}$ and $\Delta\bar{\varphi}^{out}$ follows from the identities for a single junction, in which $+0$ is substituted by L. Indeed, for voltage-dependent shifts defining outgoing charge currents we have

$$\Delta_v \bar{\varphi}^{\leftarrow}_{uc}(0) = \tfrac{1}{4}\left[3\Delta\bar{\varphi}^{\rightarrow}_{pc}(0) + \Delta\bar{\varphi}^{\leftarrow}_{uc}(L) + (\Delta\bar{\varphi}^{\rightarrow}_{us}(0) - \Delta\bar{\varphi}^{\leftarrow}_{pn}(L))\right] \tag{90}$$

$$\Delta_v \bar{\varphi}^{\rightarrow}_{pc}(L) = \tfrac{1}{4}\left[3\Delta\bar{\varphi}^{\leftarrow}_{uc}(L) + \Delta\bar{\varphi}^{\rightarrow}_{pc}(0) - (\Delta\bar{\varphi}^{\rightarrow}_{us}(0) - \Delta\bar{\varphi}^{\leftarrow}_{pn}(L))\right]. \tag{91}$$

These equations reflect the conservation of the in and out charge current. From the Supplementary Eqs.(90,91), it is apparent that 3/4 of the incoming charge current passes along the edge and 1/4 of the incoming charge current is diverted through the domain wall.

For voltage-dependent shifts defining outgoing neutral current in the polarized region and spin current in the unpolarized region we have

$$\Delta_v \bar{\varphi}^{\leftarrow}_{pn}(0) = \tfrac{1}{4}\left[3\Delta\bar{\varphi}^{\leftarrow}_{pn}(L) + \Delta\bar{\varphi}^{\rightarrow}_{us}(0) + 3(\Delta\bar{\varphi}^{\rightarrow}_{pc}(0) - \Delta\bar{\varphi}^{\leftarrow}_{uc}(L))\right] \tag{92}$$

$$\Delta_v \bar{\varphi}^{\rightarrow}_{us}(L) = \tfrac{1}{4}\left[3\Delta\bar{\varphi}^{\rightarrow}_{us}(0) + \Delta\bar{\varphi}^{\leftarrow}_{pn}(L) - 3(\Delta\bar{\varphi}^{\rightarrow}_{pc}(0) - \Delta\bar{\varphi}^{\leftarrow}_{uc}(L))\right]. \tag{93}$$

These relations reflect in and out tunneling current conservation.

Analyzing the relations Supplementary Eqs. (91, 93), we find that currents outside the domain wall of finite length are the same as in the previous settings: for single junction

(point contact), two junctions, and two junctions with allowed scattering. This property is a result of boundary conditions in the strong coupling limit.

Inside the domain wall, the chiral evolution of χ_{u2}, χ_{p2} is controlled by the average voltage shifts at their corresponding boundaries $\Delta_v\bar{\chi}_{p2}(L)$ and $\Delta_v\bar{\chi}_{u2}(0)$:

$$\Delta_v\bar{\chi}^{in}_{p2}(L) = \tfrac{1}{\sqrt{2}}\left[\Delta_v\bar{\varphi}^{\rightarrow}_{pc}(L) - \Delta_v\bar{\varphi}^{\leftarrow}_{pn}(L)\right]$$
$$= \tfrac{1}{4\sqrt{2}}\left[3\Delta_v\bar{\varphi}^{\leftarrow}_{uc}(L) + \Delta_v\bar{\varphi}^{\rightarrow}_{pc}(0) - 3\Delta_v\bar{\varphi}^{\leftarrow}_{pn}(L) - \Delta_v\bar{\varphi}^{\rightarrow}_{us}(0)\right]. \quad (94)$$

$$\Delta_v\bar{\chi}^{in}_{u2}(0) = \tfrac{1}{\sqrt{2}}\left[\Delta_v\bar{\varphi}^{\leftarrow}_{uc}(0) - \Delta_v\bar{\varphi}^{\rightarrow}_{us}(0)\right]$$
$$= \tfrac{1}{4\sqrt{2}}\left[3\Delta_v\bar{\varphi}^{\rightarrow}_{pc}(0) + \Delta_v\bar{\varphi}^{\leftarrow}_{uc}(L) - 3\Delta_v\bar{\varphi}^{\rightarrow}_{us}(0) - \Delta_v\bar{\varphi}^{\leftarrow}_{pn}(L)\right]. \quad (95)$$

If the only nonzero incoming voltage shift is $\Delta_v\bar{\varphi}^{in}_{pc}(0) = -\frac{e\sqrt{2}}{3\hbar}Vt$, and the only nonzero incoming current is $j_{pc} = \frac{2}{3}\sigma_0 V$, we find

$$\Delta_v\bar{\chi}^{in}_{p2} = -\frac{e}{12\hbar}Vt \quad (96)$$

and

$$\Delta_v\bar{\chi}^{in}_{u2} = -\frac{e}{4\hbar}Vt. \quad (97)$$

The currents carried by various modes are defined by equations:

$$j_{pc} = -\sigma_0\partial_t(q^x_{p1}\Phi_{p1} - q^x_{p2}\chi_{p2})|_{\partial_t\Phi_{p1}=0} = \sigma_0 q^x_{p2}\partial_t\chi_{p2}. \quad (98)$$

$$j_{uc} = -\sigma_0\partial_t(q^x_{u1}\Phi_{u1} + q^x_{u2}\chi_{u2})|_{\partial_t\Phi_{u1}=0} = -\sigma_0 q^x_{u2}\partial_t\chi_{u2}. \quad (99)$$

$$j_{pn} = -\sigma_0\partial_t(q^x_{p1}\Phi_{p1} - 3q^x_{p2}\chi_{p2})|_{\partial_t\Phi_{p1}=0} = 3\sigma_0 q^x_{p2}\partial_t\chi_{p2}. \quad (100)$$

$$j_{us} = -\sigma_0\partial_t(q^x_{u1}\Phi_{u1} + 3q^x_{u2}\chi_{u2})|_{\partial_t\Phi_{u1}=0} = -3\sigma_0 q^x_{u2}\partial_t\chi_{u2}. \quad (101)$$

Therefore, the average currents along the domain wall are

$$j^{DW}_{pc} = \tfrac{\sigma_0}{12}V \quad (102)$$
$$j^{DW}_{pn} = \tfrac{\sigma_0}{4}V \quad (103)$$
$$j^{DW}_{uc} = \tfrac{\sigma_0}{4}V \quad (104)$$
$$j^{DW}_{us} = \tfrac{3\sigma_0}{4}V. \quad (105)$$

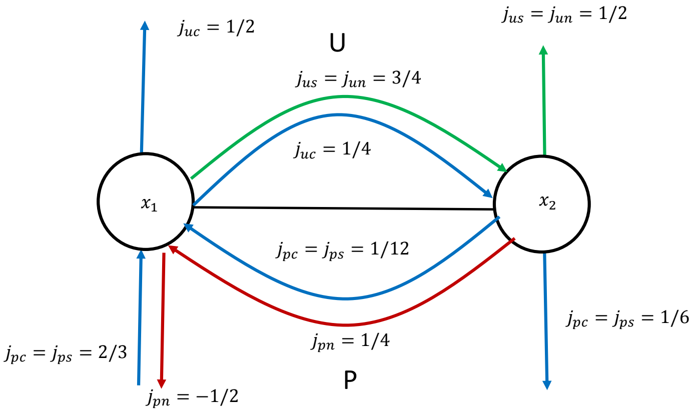

Supplementary Figure 10. **The current flow for current $j_c = 2/3$ injected from the polarized into the unpolarized region.** Values of currents are shown in units of $\sigma_0 V$. Charge currents in the polarized and unpolarized regions are shown in blue. In the polarized region, the charge current and the spin current coinside. The neutral current in polarized region, describing the difference of currrents carried by two quasiparticle modes, is shown in red. Spin currents in the unpolarized region, corresponding to the difference of currents carried by spin up and spin down modes in there, are shown in green.

I. Summary of charge, spin and neutral currents in the absence of spin flip processes

We now summarize our results, see Supplementary Fig. 10. We use indices 1 and 2 to describe the incoming (index *in*) and outgoing (index *out*) currents flowing along sample edges through the junctions at x_1 and x_2, where edge channels at the boundary of the sample are coupled to the channels propagating inside the domain wall. The currents (index DW) inside the domain wall flow from left to right (+sign) and right to left (-sign). For the currents outside the domain wall, we then have

$$j_{pc}^{in,1} = \frac{2}{3}\sigma_0 V, \qquad j_{uc}^{out,1} = \frac{1}{2}\sigma_0 V$$

$$j_{us}^{in,1} = 0 \qquad j_{pn}^{out,1} = -\frac{1}{2}\sigma_0 V \tag{106}$$

$$j_{pn}^{in,2} = 0 \qquad j_{pc}^{out,2} = \frac{1}{6}\sigma_0 V \tag{107}$$

$$j_{uc}^{in,2} = 0 \qquad j_{us}^{out,2} = \frac{1}{2}\sigma_0 V, \tag{108}$$

where the upper index 1 corresponds to the incoming (outgoing) currents at the junction x_1 and the upper index 2 corresponds to the incoming (outgoing) currents at the junction x_2. For the currents inside the domain wall, we have

$$\begin{aligned} j_{uc}^{DW} &= \frac{1}{4}\sigma_0 V & j_{us}^{DW} &= \frac{3}{4}\sigma_0 V \\ j_{pc}^{DW} &= \frac{1}{12}\sigma_0 V & j_{pn}^{DW} &= \frac{1}{4}\sigma_0 V \end{aligned} \tag{109}$$

It is easy to see that at the each junction the charge current is conserved and so are the spin currents in the unpolarized liquid and the neutral currents in the polarized liquid. Also, the sum of spin projections of incoming electrons is equal to the sum of spin projections of outgoing electrons at junctions x_1 and x_2. As we discussed in subsection H, Supplementary Eqs.(108) hold for general case of the domain wall of finite length.

The case of an incoming charge current from the unpolarized liquid flowing into the polarized liquid, is described by similar equations. Results for charge currents are given then by Supplementary Eq.(108) with a permutation $p \leftrightarrow u$. For the spin and neutral currents the resultant equations are given by Supplementary Eq.(108) with a simultaneous permutation $p, n \leftrightarrow u, s$. Similarly, there are conservation laws for charge currents, spin currents in the unpolarized phase, neutral currents in the polarized phase at the x_1 and x_2 junctions on the edge of the sample. The domain wall in both cases, when the current injected from the p phase into the u phase, and when the current injected from the u phase into the p phase, can be described by the ratio $i_{DW} = 1/3$.

J. Effect of spin flip processes

So far we considered tunneling and/or scattering between modes assuming that no spin flips are allowed. Experiment, however, clearly demonstrates substantial role of spin-flip processes associated with nuclear spins. Such spin-flip processes due to hyperfine interactions can occur in the vicinity of the domain wall, where it is possible to match small nuclear spin splitting and electron energy splitting near the crossing of composite fermion levels.

We will model partial spin flip by assuming that nuclear spin flips may result in an admixture of polarized and unpolarized propagating density modes inside the domain wall. In the model discussed above, tunneling of like spins is defined by tunneling Hamiltonian Supplementary Eq. (72) that completely determines the electronic mode Φ_1. In the absence

of spin flip processes, the χ_2 quasiparticle modes are free chiral propagating modes, which correspond to uncoupled propagation of χ_2 modes with opposite spins, as given by Supplementary Eqs. (81, 82). In the presence of spin flips in the domain wall, the χ_{2u} mode, which is a superposition of a charge and a spin mode, and the χ_{2p} mode, which is a superposition of a charge and a neutral mode, become coupled. In particular, the incoming χ_{2p} mode at the junction $x = x_2 = L$ that propagates as charge and neutral mode towards $x = x_1 = 0$, is partially reflected into u phase through junction $x_2 = L$ as χ_{2u} mode with probability r, and partially continues propagation towards $x_1 = 0$ with probability $1 - r$. The χ_{2u} charge and spin modes incoming at junction $x = x_1 = 0$, are reflected with probability r as χ_{2p} modes and partially propagate with probability $1 - r$ towards $x = x_2 = L$ as χ_{2u} modes. These processes are summarized as boundary conditions

$$\Delta_v\bar{\varphi}^{\leftarrow}_{uc}(L) - \Delta_v\bar{\varphi}^{\rightarrow}_{us}(L) = (1-r)\left[\Delta_v\bar{\varphi}^{\leftarrow}_{uc}(0) - \Delta_v\bar{\varphi}^{\rightarrow}_{us}(0)\right] + r\left[\Delta_v\bar{\varphi}^{\rightarrow}_{pc}(L) - \Delta_v\bar{\varphi}^{\leftarrow}_{pn}(L)\right], \tag{110}$$

$$\Delta_v\bar{\varphi}^{\rightarrow}_{pc}(0) - \Delta_v\bar{\varphi}^{\leftarrow}_{pn}(0) = (1-r)\left[\Delta_v\bar{\varphi}^{\rightarrow}_{pc}(L) - \Delta_v\bar{\varphi}^{\leftarrow}_{pn}(L)\right] + r\left[\Delta_v\bar{\varphi}^{\leftarrow}_{uc}(0) - \Delta_v\bar{\varphi}^{\rightarrow}_{us}(0)\right]. \tag{111}$$

At $r = 0$, these boundary conditions coincide with boundary conditions Supplementary Eqs. (81, 82). Solving these equations together with Supplementary Eqs. (83, 84) for the sole source of non-zero incoming current $j_{pc} = \frac{2}{3}\sigma_0 V$ results in relation

$$(3-r)\Delta_v\bar{\varphi}^{\rightarrow}_{pc}(L) = (1-r)\Delta_v\bar{\varphi}^{\leftarrow}_{uc}(0). \tag{112}$$

The charge, spin and neutral currents outside the domain wall in the presence of spin flips are modified as follows, compared to Supplementary Eq. (108):

$$j^{in,1}_{pc} = \frac{2}{3}\sigma_0 V, \qquad j^{out,1}_{uc} = \frac{3-r}{3(2-r)}\sigma_0 V \tag{113}$$

$$j^{in,1}_{us} = 0 \qquad j^{out,1}_{pn} = -\frac{1-r}{2-r}\sigma_0 V \tag{114}$$

$$j^{in,2}_{pn} = 0 \qquad j^{out,2}_{pc} = \frac{1-r}{3(2-r)}\sigma_0 V \tag{115}$$

$$j^{in,2}_{uc} = 0 \qquad j^{out,2}_{us} = \frac{1-r}{2-r}\sigma_0 V, \tag{116}$$

where we used the same notation as in Supplementary Eq.(108), with index 1 corresponding $x = 0$ and index 2 corresponding $x = L$. Therefore, we see that allowing all spin-flip

processes in electron tunneling and scattering inside the domain wall, $r = 1$, leads to the absence of current through the domain wall, so that the current is flowing only along the sample edges, with the Hall resistance quantized to $3/2\ h/e^2$ and zero longitudinal resistance. This result is naturally expected, as $r = 1$ essentially describes the absence of the domain wall. The domain wall current and currents flowing along the edge are the functions of the spin-flip probability r. The ratio $i_{DW} = I_{DW}/I$ changes continuously between 1/3 for $r = 0$ to zero for $r = 1$, where I_{DW} is the current diverted from the edge through the domain wall, and I is the injected current.

---

[1] C. W. J. Beenakker, *Edge channels for the fractional quantum Hall effect*, Phys. Rev. Lett. **64**, 216 (1990).

[2] Luis Brey, *Edge states of composite fermions*, Phys. Rev. B **50**, 11861 (1994).

[3] Xiao-Gang Wen, *Topological orders and edge excitations in fractional quantum Hall states*, Adv. Phys. **44**, 405 (1995).

[4] C. L. Kane, Matthew P. A. Fisher, and J. Polchinski, *Randomness at the edge: Theory of quantum Hall transport at filling* $\nu = 2/3$, Physical Review Letters **72**, 4129 (1994).
